# The Potential of Sewage Sludge to Predict and Evaluate the Human Chemical Exposome

**DOI:** 10.1021/acs.estlett.1c00848

**Published:** 2021-11-12

**Authors:** Ruben Gil-Solsona, Maria-Christina Nika, Mariona Bustamante, Cristina M. Villanueva, Maria Foraster, Marta Cosin-Tomás, Nikiforos Alygizakis, Maria Dolores Gómez-Roig, Elisa Llurba-Olive, Jordi Sunyer, Nikolaos S. Thomaidis, Payam Dadvand, Pablo Gago-Ferrero

**Affiliations:** † Department of Environmental Chemistry, Institute of Environmental Assessment and Water Research − Severo Ochoa Excellence Center (IDAEA), Spanish Council of Scientific Research (CSIC), Jordi Girona 18-26, Barcelona 08034, Spain; ‡ Laboratory of Analytical Chemistry, Department of Chemistry, National and Kapodistrian University of Athens, Panepistimiopolis Zografou, Athens 15771, Greece; § ISGlobal, Barcelona 08003, Spain; ∥ Universitat Pompeu Fabra (UPF), Barcelona 08003, Spain; ⊥ CIBER Epidemiología y Salud Pública (CIBERESP), Madrid 28029, Spain; # IMIM (Hospital del Mar Medical Research Institute), Doctor Aiguader 88, Barcelona 08003, Spain; ∇ PHAGEX Research Group, Blanquerna School of Health Science, Universitat Ramon Llull (URL), Carrer de Padilla, 326, Barcelona 08025, Spain; ○ Department of Human Genetics, Research Institute of the McGill University Health Center, McGill University, 845 Sherbrooke St W, Montreal, Quebec H3A 0G4, Canada; ◆ BCNatal − Barcelona Center for Maternal Fetal and Neonatal Medicine (Hospital Sant Joan de Déu and Hospital Clínic), University of Barcelona, Esplugues de Llobregat, Passeig de Sant Joan de Déu, 2, Barcelona 08950, Spain; α Maternal and Fetal Medicine Unit, Obstetrics and Gynecology Department, Sant Pau University Hospital, C. de Villarroel, 170, Barcelona 08036, Spain; β Development Network (SAMID), RD16/0022/0015, Instituto de Salud Carlos III, Av. de Monforte de Lemos, 5, Madrid 28029, Spain

## Abstract

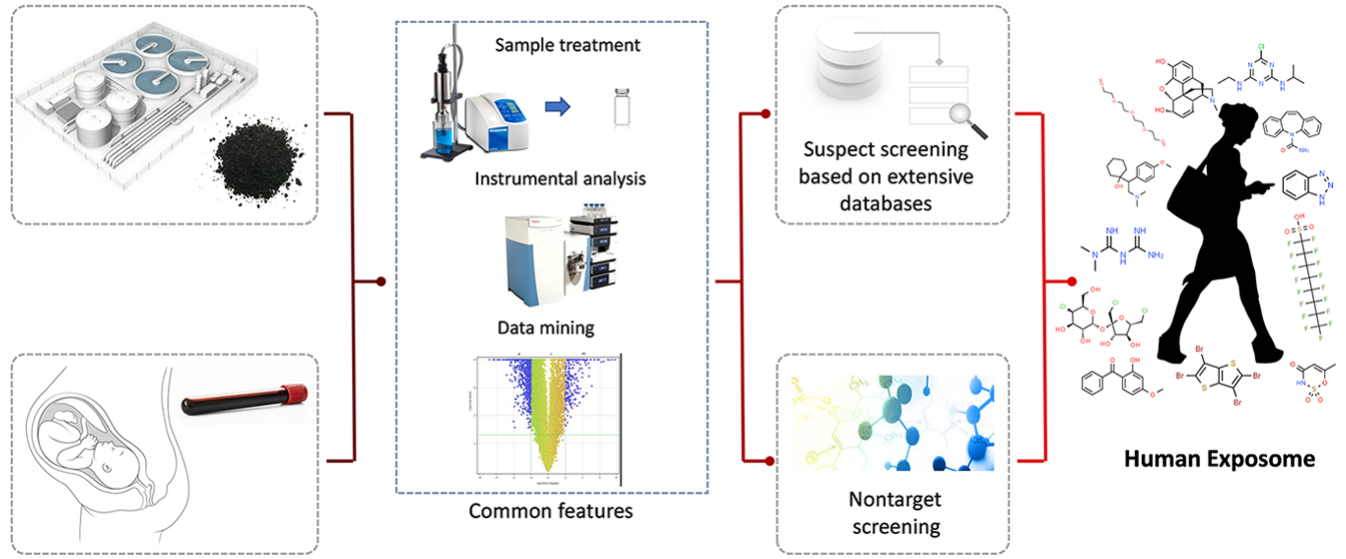

Chemicals are part of our daily lives, and we are exposed to numerous chemicals through multiple pathways. Relevant scientific evidence contributing to the regulation of hazardous chemicals require a holistic approach to assess simultaneous exposure to multiple compounds. Biomonitoring provides an accurate estimation of exposure to chemicals through very complex and costly sampling campaigns. Finding efficient proxies to predict the risk of chemical exposure in humans is an urgent need to cover large areas and populations at a reasonable cost. We conducted an exploratory study to characterize the human chemical exposome in maternal blood and placenta samples of a population-based birth cohort in Barcelona (2018–2021). Ultimate HRMS-based approaches were applied including wide-scope target, suspect, and nontarget screening. Forty-two chemicals were identified including pesticides, personal care products, or industrial compounds, among others, in the range of ng/mL and ng/g. In parallel, sewage sludge from the wastewater treatment plants serving the residence areas of the studied population were also screened, showing correlations with the type and concentrations of chemicals found in humans. Our findings were suggestive for the potential use of sewage sludge as a proxy of the human exposure and its application in early warning systems to prevent bioaccumulation of hazardous chemicals.

## Introduction

The presence of chemicals in our everyday environment plays a prominent role in the development of complex diseases, especially chronic ones.^1−3^ Over the last decades, there has been a considerable increase in cases of allergies, infertility, impaired brain development in children, various types of cancer, or even neurological disorders that can be related to environmental exposures.^4−8^ However, it is challenging to develop compelling evidence that certain chemicals present in the environment cause specific adverse health effects. Furthermore, the increasing addition of new chemicals in the market could be worsening these situations. One of the principal factors of this difficulty is the lack of knowledge on the presence and bioaccumulation of organic chemicals; the chemical *exposome*, defined as the totality of environmental chemical exposures from birth onward,^9^ is far from being completely understood. Therefore, scientific evidence is essential to obtain a holistic understanding of both the presence of new chemicals and their potential effects on human health.

Understanding the chemicals we are exposed to in our workplace and daily lives remains one of the main challenges regulators face when addressing chemicals of concern. Biomonitoring provides an accurate method to estimate the exposure and bioaccumulation of organic chemicals through the direct measurement of the chemicals in the relevant biofluids and tissues. The analytical strategies (based on target screening) that have been commonly applied in human biomonitoring are biased because they only consider a very limited portion of the existing universe of chemicals.^10−13^ Advances in high-resolution mass spectrometry (HRMS) have opened windows of opportunity for the identification of previously unknown chemicals of concern. The emergence of large chemical repositories, containing MS/HRMS data (e.g., Massbank,^14^ Massbank of North America,^15^ Metlin,^16^ HMDB,^17^ mzCloud^18^), as well as tools for wide-scope suspect screening (e.g., NORMAN DSFP^19^) or automatic annotation of unknowns (e.g., SIRIUS4,^20^ MetFrag^21^), have provided an unprecedented possibility to perform large-scale investigations. In the framework of projects and initiatives related to human exposure (such as HEALS,^22^ HBM4 EU,^23^ EXPOsOMICS,^24^ Project VIVA,^25^ ATHLETE,^26^ or FIGO^27^), few studies have successfully applied these strategies, identifying potential chemical exposures in human samples.^28−31^

However, human biomonitoring campaigns are very complex since they (I) deal with logistical constraints, (II) use invasive techniques and imply ethical issues, and (III) impose considerable economic burden. These factors make it difficult to cover large geographical areas and reach large populations. Finding efficient proxies to predict the risk of chemical exposure in humans is an urgent need to cover a significant fraction of the population and specific environments. In this context, sewage epidemiology, currently used to estimate the behavior of a large-scale population regarding the consumption of specific substances (illicit drugs,^32^ pharmaceuticals,^33^ and plastisizers^34^) or to predict COVID-19 outbreaks,^35^ can play an important role.

In this work, we conducted an exploratory study using advanced HRMS-based strategies (wide-scope target, suspect, and nontarget) to extensively characterize the human chemical *exposome* in maternal blood (*N* = 10) and placenta samples (*N* = 9) from 19 individuals available from the population-based BiSC (Barcelona Life Cohort Study, http://projectebisc.org) mother–child cohort. In parallel, sewage sludge (the residue of wastewater treatment) from the wastewater treatment plants (WWTP) serving the residence areas of the studied population was also collected and screened using compatible methodologies. As such, this study is a proof of concept for the evaluation of the potential use of sewage sludge as a proxy of the human chemical exposome and its application in early warning systems to prevent the bioaccumulation of hazardous chemicals, without the need of conducting extensive biomonitoring campaigns. The similarity of human and sewage sludge samples was evaluated in terms of presence/absence of xenobiotics and overall matrix composition using nontarget approaches.

## Materials and Methods

### Study Population and Sample Collection

Recruitment occurred from October 2019 until March 2020 in three main tertiary hospitals of Barcelona, Spain. Exogenous chemicals enter the human body, are distributed by blood, and finally reach different tissues, where they can be heterogeneously bioaccumulated depending on tissue, species, and chemical.^36^ The pilot study included 10 blood samples (serum and plasma) from mothers obtained at the time of delivery and nine placentas from a total of 19 pregnant women. Maternal blood was collected in different tubes and centrifuged to obtain serum and plasma. Placental biopsies (0.4 mm × 0.4 mm × full thickness) were taken from two different quadrants, rinsed in saline, snap frozen in liquid nitrogen, and stored at −80 °C until analysis. A fraction of 200 mg from the fetal villi of one of these biopsies was analyzed. Additional details of the cohort and the mother–child pairs can be found in the SI.

Sewage sludge was collected from the only two WWTPs, from September 2019 to March 2020, which serve the areas of the whole Barcelona metropolitan area. Sludge samples (*n* = 6) consisting of 250 g were collected each 4 months, as sewage sludge composition undergoes very small variations over time, freeze dried, and stored at −20 °C until extraction.

### Chemicals and Analysis

Details about the chemicals and reagents and extensive descriptions of the sample treatment protocols are provided in the SI. Briefly, aliquots of plasma and serum (150 μL) were deproteinized with acetonitrile (ACN), and the supernatant was collected for analysis. Placenta samples (200 mg) were extracted with a mixture of ACN, citric acid buffer, and zirconium beads in a tissuelyser. Then, a SPE cleanup step with mixed mode cartridges was carried out as described elsewhere.^37,38^ Sewage sludge (1 g) was extracted with a digital sonifier using ACN and water, and further cleanup and preconcentration steps were conducted through SPE. Procedural blanks (*n* = 5 for each matrix) were processed following the same protocols, in order to assess potential contaminations.

Instrumental analysis was carried out using a MaXis Impact Q-ToF instrument (Bruker Daltonics) and a Q-Exactive Q-Orbitrap instrument (Thermo Scientific) coupled to UHPLC. Acquisition was performed using both data-independent and data-dependent modes (DIA and DDA, respectively). The scheme shown in Figure S1 summarizes the followed workflow, and full details can be also consulted in the SI.

### Target, Suspect, and Nontarget Screening Strategies

Wide-scope target analysis was performed for more than 2300 organic chemicals, including pharmaceuticals, illicit drugs, doping compounds, herbicides, surfactants, and other industrial chemicals as well as transformation products following the procedure described elsewhere.^39^ The complete list of chemicals as well as the analytical evaluation procedure is available in the aforementioned publication.

For extended suspect screening (SI), DIA data from a Q-Exactive instrument was transformed from a proprietary (∗.raw) to generic (∗.mzML) format and uploaded in the DSFP platform^19^ along with calculated retention time indexes of calibrant substances.^40^ The screening included 7586 chemicals (EXPHRMSMSAVAL list) with a wide range of uses and physicochemical properties (full list available in the SI spreadsheet). Experimental MS/MS information for all substances was available. The identification of the chemicals was first based on mass accuracy, isotopic fit, and an advanced LC retention time prediction model combined with the inspection of MS/MS fragments. Then, tentative identifications for exogenous compounds were verified or discarded manually checking the DDA data. Other metadata linked with commercial indicators was used in some cases as described elsewhere.^41,42^

For nontarget screening, DDA data from Q-Exactive was transformed to generic format (∗.mzML) and data mined in MZMine2.^43^ Peak picking was performed only for those features with one MS/MS spectrum acquired in, at least, one sample of the whole data set (in order to avoid the inclusion of instrumental noise as much as possible). The feature list was then exported to SIRIUS4.^20^ Both molecular formula and compound annotations were obtained, based on the information on accurate mass, isotopic pattern, and MS/MS spectra. Chemicals with observed negative mass defect, suggesting halogenated species and therefore of anthropogenic origin, and not previously identified in the target and suspect analysis were further explored in a nontarget manner (SI), using different spectral online databases (e.g., MassBank,^14^ MoNa,^15^ or Metlin^16^) and automatic annotation tools (e.g., SIRIUS^20^).

In all cases, three procedural blanks for serum, placenta, and sludge were carefully assessed to avoid false positives. Reference standards were purchased for the confirmation of most tentatively identified compounds, including all listed in Table 1 with a level of confidence 1. When the standard was not available, chemicals remained with a lower confidence level.^44^ Concentrations for the newly confirmed chemicals and the tentatively identified ones were estimated^45,46^ (SI-7).

**Table 1:** Details on 43 Chemicals Identified in Human Samples

Identity	Common use	Strategy	Confidence level^a^	Serum: Detection (%)/Conc. range (ng/mL)	Placenta: Detection (%)/Conc. range (ng/g)	Sludge: Detection (%)/Conc. range (ng/g)
Tris(chloropropyl)phosphate	Flame retardant	Nontarget	Level 1	100/(0.2–15)	22/(n.d. – 1.4)	100/(3.6–13)
1,7-Dimethylxanthine	Food component	Suspect	Level 2	50/(n.d. – 0.05)	0 (−)	50/(n.d. – 2.2)
Theobromine	Food component	Target	Level 1	100/(0.01–25)	78/(n.d. – 542)	17/(n.d. – 2.6)
Theophylline	Food component	Target	Level 1	90/(n.d. – 3.8)	89/(n.d. – 540)	33/(n.d. – 2.2)
Lenticin	Food component	Suspect	Level 2	100/(3.7–13)	78/(n.d. - 211)	83/(n.d.- 13)
Caffeine	Food component	Target	Level 1	100/(0.6–45)	78/(n.d. – 840)	100/(0.9–4.8)
Endothall	Herbicide	Target	Level 1	50/(n.d. – 0.3)	0 (−)	83/(n.d. – 16)
Denatonium	Industrial	Suspect	Level 1	0 (−)	44/(n.d. – 44)	67/(n.d. – 2.3)
Benzotriazole	Industrial	Suspect	Level 1	0 (−)	44/(n.d. – 3.2)	100/(0.4–0.6)
Dibenzylamine	Industrial	Suspect	Level 2	0 (−)	44/(n.d. – 99)	50/(n.d. – 24)
Tributyl phosphate	Industrial	Suspect	Level 2	100/(0.2–1.2)	11/(n.d. – 0.4)	100/(0.6–10)
Triphenyl phosphine oxide	Industrial	Suspect	Level 1	0 (−)	70/(n.d. – 8.3)	100/(0.3–0.5)
Triphenyl phosphate	Industrial	Suspect	Level 1	100/(0.6–1.6)	0 (−)	10/(n.d. – 0.01)
2-hydroxybenzothiazole	Industrial	Suspect	Level 1	0 (−)	78/(n.d. – 34)	100/(0.2–1.2)
2-benzothiazolesulfonic acid	Industrial	Nontarget	Level 1	0 (−)	78/(n.d. – 2.3)	84/(n.d. – 0.02)
DEET	PCP/Pesticide	Target	Level 1	40/(n.d. – 0.4)	0 (−)	100/(0.9–13)
N,N-Dimethyldodecylamine-N-oxide	PCP	Suspect	Level 2	90/(n.d. – 0.8)	44/(n.d. – 0.2)	100/(0.1–1.2)
Panthenol	PCP	Suspect	Level 1	0 (−)	44/(n.d. – 21)	17/(n.d. – 1.4)
Bis-(2-ethylhexyl)amine	PCP	Suspect	Level 2	60/(n.d. – 27)	22/(n.d. – 48)	100/(>1000)
N-(2-hydroxyethyl)-Tetradecanamide	PCP	Suspect	Level 2	0 (−)	100/(0.03–1.5)	100/(0.6–14)
Lauryl diethanolamine	PCP	Suspect	Level 1	90/(n.d. – 5.2)	67/(n.d. – 3.2)	100/(0.4–0.5)
Benzododecinium	PCP	Target	Level 1	60/(n.d. – 86)	67/(n.d. – 118)	100/(326–664)
PFOS	PFAS	Suspect	Level 1	100/(0.08–0.3)	55/(n.d. – 34)	100/(4.1–10)
PFHpS	PFAS	Suspect	Level 1	0 (−)	33/(n.d. – 2.9)	0 (−)
PFBS	PFAS	Suspect	Level 1	0 (−)	11/(n.d. – 0.8)	0 (−)
6:2 FTS	PFAS	Suspect	Level 1	0 (−)	11/(n.d. – 1.4)	50/(n.d. – 0.2)
PFHxS	PFAS	Suspect	Level 1	20/(n.d. – 0.02)	0 (−)	0 (−)
Bupivacaine	PhAC	Suspect	Level 2	0 (−)	100/(0.8–126)	0 (−)
Ciprofloxacin	PhAC	Suspect	Level 1	0 (−)	11/(n.d. – 7.3)	0 (−)
Amoxicillin	PhAC	Suspect	Level 1	0 (−)	11/(n.d. – 6.6)	0 (−)
Indole-3-acetic acid	Plant hormone	Suspect	Level 1	100/(2.8–8.9)	78/(n.d. – 1.4)	100/(0.5–2.6)
Dibutyl phthalate	Plastic additive	Target	Level 2	80/(n.d. – 9.6)	100/(11.1–23)	100/(27–63)
Monobuthyl phthalate	Plastic additive	Suspect	Level 2	30/(n.d. – 2.5)	89/(n.d. – 10)	100/(9.2–15)
4-Ethoxyethylbenzoate	Plastic additive	Suspect	Level 2	100/(11–69)	100/(51.5–186)	100/(70.3–96)
1,3-Diphenylguanidine	Plastic additive	Suspect	Level 2	70/(n.d. – 28)	0 (−)	100/(16–79)
Nicotine	Stimulant	Suspect	Level 1	0 (−)	67/(n.d. – 15)	100/(2.4–70)
Cotinine	Stimulant TP	Target	Level 1	20/(n.d. – 56)	33/(n.d. – 7.5)	67/(n.d. – 587)
Acesulfame	Sweetener	Target	Level 1	20/(n.d. – 0.5)	11/(n.d. – 8.1)	0 (−)
Methylsulfonyl-1,3-thiazol-2-ylamine	Unknown	Nontarget	Level 3	0/(−)	22/(n.d. – 6.2)	0 (−)
1-Benzothiophene-2-Sulfonamide	Unknown	Nontarget	Level 3	0 (−)	89/(n.d. – 19)	17/(n.d. – 0.1)
Benzophenone	UV filter	Suspect	Level 1	10/(n.d. – 0.07)	78/(n.d. – 3.4)	100/(0.2–7.7)

a According to Schymanski et al.^41^. PCP, personal care products; PhAC, pharmaceutical active compounds; PFAS, per- and polyfluoroalkyl substances; n.d., not detected.

## Results and Discussion

### Extensive Screening of Organic Chemicals in Blood and Placenta

In total, 42 chemicals were identified using the three various workflows (target, suspect, and nontarget analysis) in the selected serum and placenta samples (18 in common, 25 in serum, 35 in placenta). Out of those chemicals, 28 were confirmed with reference standards, and 14 remained as tentatively identified. Of the 42 reported chemicals, nine were identified via wide-scope target analysis and 29 as a part of the suspect analysis, including pharmaceuticals, food-derived chemicals, herbicides, insect repellents, and a variety of industrial chemicals. Additionally, features with a negative mass defect and Cl and Br isotope information, indicator of an exogenous origin and potentially hazardous, were prioritized for further identification via nontarget analysis. Despite this restrictive criterion, four additional chemicals not included in any of the previous list were tentatively identified including tris(chloropropyl)phosphate, 2-benzothiazolesulfonic acid, methylsulfonyl-1,3-thiazol-2-ylamine, and 1-benzothiophene-2-sulfonamide, classified as flame retardants (and other industrial uses). For all the identified chemicals, their identity, common uses, types of strategy that led to their identification, confidence levels, frequencies of detection, and (semi)quantitative concentration ranges are summarized in Table 1. Limits of detection (LODs) were in the range of 0.01–1.7 ng/mL in serum and 0.01–4.9 ng/g in placenta. The analytical evidence that led to the identifications are provided in Table S2. The results obtained in plasma and serum were identical in qualitative and quantitative terms. Therefore, in this study, we show only serum results.

The presence of the exogenous chemicals in biofluids and tissues could be explained by multiple types of exposure, mainly inhalation, ingestion, or dermal contact.^47,48^ In exceptional cases, such as for some drugs, it may be by injection, as is the case with Bupivacaine (used as epidural and intradural anesthesia).^49^ This chemical was found in all placental samples (since all the mothers used local anesthesia during delivery). Nicotine-detected levels (up to 15 ng/g in placenta) and its main metabolite Cotinine (up to 56 ng/mL in serum and 7.5 ng/g in placenta) showed a certain active or passive exposure to tobacco for 67% of the individuals. This percentage is in line with previous studies that in some cases have reported higher levels of nicotine in placenta (up to 108 ng/g)^50^ and plasma (up to 19.6 ng/mL of nicotine and 97.1 ng/mL of cotinine)^51^ of smokers. Low levels (up to 0.4 ng/mL) of the insect repellent N,N-diethyl-3-methylbenzamide (DEET) were also determined in 40% of the serum samples. This chemical is commonly used in our study area, and its presence has been previously reported in both human (at similar levels, ≈3.2 ng/mL in maternal blood)^52^ and environmental samples including air and drinking water.^53^ High levels of the disinfectant Benzododecinium (up to 118 ng/g (placenta) and 86 ng/mL (serum)) were detected in greater than 60% of the samples. This chemical is present in products such as Sterinol, widely used as disinfectant before and during the wake of the COVID-19 pandemic^46^ and have been found to cross skin with intensive usage, reaching levels in plasma of up to 141.2 ng/L after an intensive five day usage.^54^ Additional chemicals used in personal care products were also present in human matrices (Table 1) including N,N-dimethyldodecylamine-N-oxide, bis(2-ethylhexyl)amine, N-(2-hydroxyethyl)-tetradecanamide, and lauryl diethanolamine. Furthermore, we detected various plastic additives such as 1,3-diphenylguanidine, dibutyl phthalate, and its metabolite monobutyl phthalate. Additionally, high levels (up to 186 ng/g in placenta and 69 ng/mL in serum) were determined for 4-ethoxyethylbenzoate, which is used in food contact plastics (e.g., polypropylene). Also, a wide variety of industrial compounds such as benzotriazole, dibenzylamine, tributyl phosphate, triphenylphospine oxide, benzothiazole derivatives, or the flame retardant tris(chloropropyl)phosphate were identified. Six PFAS (for which various routes of exposure have been recognized^55,56^) were found at low levels in both matrices, except for PFOS, where concentrations up to 34 ng/g were detected in the placenta.

Significant differences were observed in the distribution of the chemicals in these matrices. In general, compounds with high LogP (Table S2) showed higher levels in placenta compared to serum, indicating bioaccumulation. On the contrary, polar compounds found in serum at low concentrations were not detected in the placenta (e.g., dimethylxanthine, 1,3-diphenylguanidine, endothall, or DEET).

### Human Samples and Sewage Sludge: Similarities and Differences in Their Chemical Profiles

Chemicals are retained in sewage sludge according to their chemical abundance, bioaccumulation potential, and environmental persistence.^57^ We hypothesize that the presence of xenobiotics in urban sludge could be indicative of their bioaccumulation in humans. Under this premise, a comparison of the evaluated human samples and sewage sludge from WWTP serving the area of residence of those individuals (collected during the same period) was performed in terms of prevalence and levels of xenobiotics, physicochemical properties, and overall chemical composition (feature distribution).

More than 80% of the compounds identified in the human samples (serum or placenta) were present in the sludge (LODs were in the range 0.09–11 ng/g). The overlap was 67% in the case of placenta and 55% for serum. Chemicals not detected in sludge (Acesulfame, Bupivacaine, Ciprofloxacin, Amoxicillin, methylsulfonyl-1,3-thiazol-2-ylamine, PFAS) were all determined at very low levels (0.02–8 ng/g) and low frequency of appearance (mainly less than 30%) in the human samples. Also, those chemicals were mainly highly biodegradable except the PFASs and Acesulfame, whose LODs in sludge were not sufficient for such low levels.

**Figure 1:**
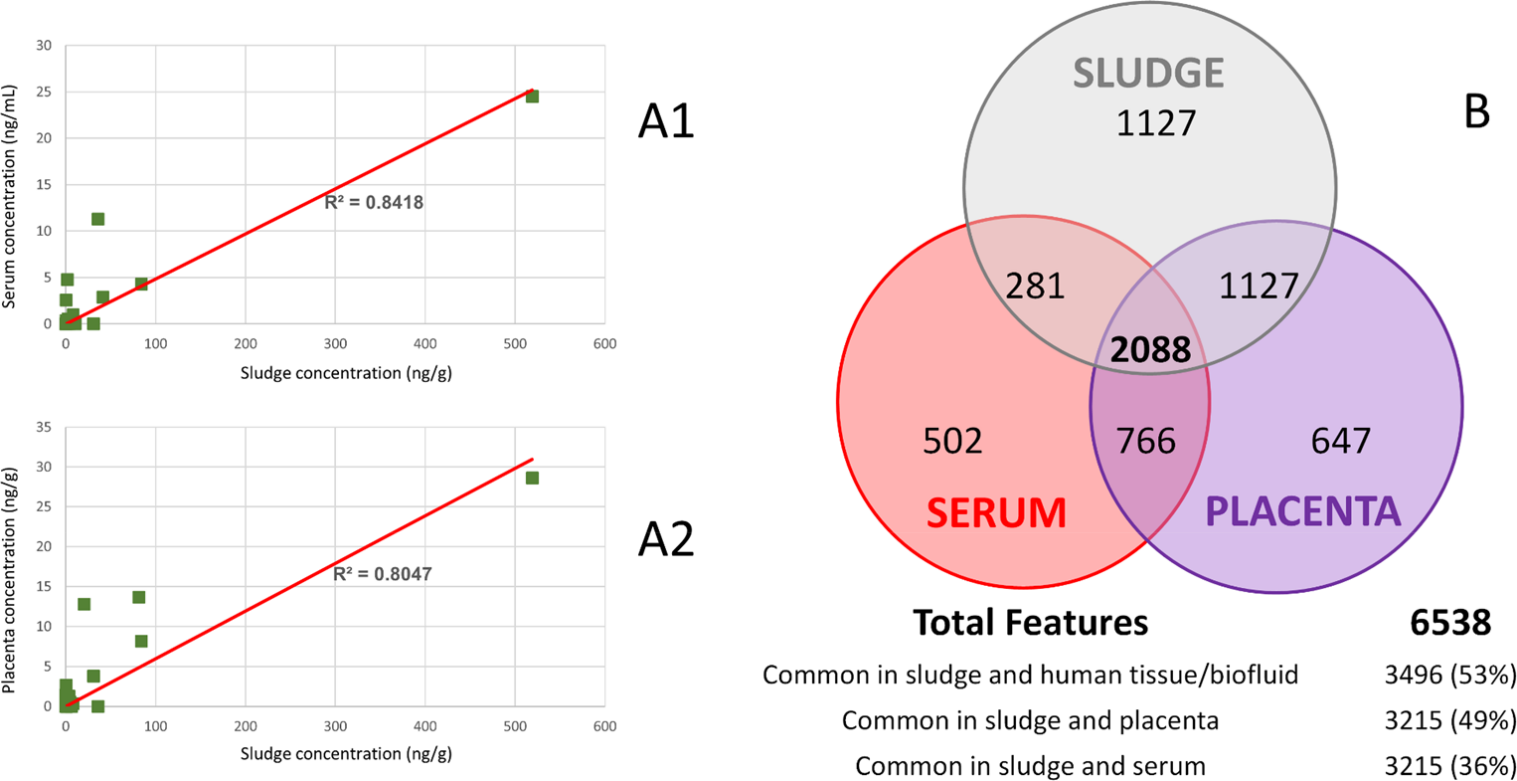
(A) Correlation between the mean concentration levels found in sludge and serum (A1) and placenta (B1). Stimulants (Caffeine, Theobromine, and Theophylline) were excluded due to their recent intake by human individuals before sample collection. (B) Venn diagram representing all features (combining positive and negative ionization modes) in common between human matrices and sludge. Only features with available MS/MS data were considered.

A certain correlation (R^2^ > 0.8) was observed between the levels found in the human samples and those found in the sludge, as can be observed in Figure 1A (R^2^ > 0.6 if excluding Benzododecinium, the chemical present at the highest levels). Although the number of compounds evaluated is small and, in some cases, the reported levels are semiquantitative, the graphic reflects the potential link between the concentrations in sludge and those found in humans.

Obviously, sewage sludge may contain a large number of chemicals that were not detected in human samples. Those compounds, especially the ones with high toxicity potential, poor biodegradability, and high concentrations, would require an additional assessment in larger cohorts, since they are candidates to bioaccumulate and result in potential hazardous effects.

Apart from the xenobiotics identified through target and nontarget strategies, ≈3500 additional common features were determined out a total of 6500 (54%). It is noteworthy that only features with MS/MS spectra available after acquisition in DDA were considered to avoid the inclusion of instrumental noise. More than 2000 features were common in sludge, serum, and placenta, 1000 more between placenta and sludge, and 300 between serum and sludge. This reveals a certain similarity between the matrices, as can be seen in the Venn diagram (Figure 1B). These common features include potential xenobiotics, for which the identity is still unknown, and mainly endogenous biomolecules (mostly lipid-like and protein-like, as it can be observed in the van Krevelen diagram (Figure S2)) among the evaluated matrices.

### Perspectives on Use of Sewage Sludge as a Proxy to the Human Body to Explore Human Exposure to Organic Chemicals

The experimental results obtained in this proof of concept study showed that the screening of sludge can be useful to obtain information on the risk of certain substances to bioaccumulate (or pseudobioaccumulate) in humans. Optimizing this approach could allow covering a large part of the population at a small cost, limiting complex biomonitoring campaigns to cases with specific objectives. The existing nonexperimental approaches usually consider the persistence, general bioaccumulation potential, and toxicity. Using sewage epidemiology (understood as the analysis of sludge), we add to the equation the current chemical production rates, consumption, and, above all, the individual behavior of chemicals in real world biological systems.

We hypothesize that sewage sludge can offer a clearer picture of the bioaccumulation of persistent chemicals than other matrices such as wastewater since, due to its carbon- and lipid-rich structure (as mentioned before), it can act as a proxy to the human body. Moreover, the concentrations of organic chemicals undergo very small variations over time (it is equivalent to an integrated sample of several days).^58,59^ Nevertheless, excretion is not the only way for chemicals to reach urban WWTPs. Minor industrial effluents and other inputs also influence the composition of sludge. The addition of those compounds with a nonstrictly urban origin will help to obtain a more comprehensive overview of what chemicals a community may be exposed to through other pathways and evaluate potential future exposures. Our results are reinforced by studies based on data extracted from the literature. A set of chemicals detected in the USA representative of biological specimens from humans (performed by the Centers for Disease Control and Prevention^60^) and municipal sewage sludge (U.S. Environmental Protection Agency’s national sewage sludge surveys^61^), revealed important (70%) overlap (out of the 52 compounds evaluated using basic target strategies).^57^

As in any study focused on the analysis of organic chemicals, the covered chemical space is limited. In this work, we implemented methodologies for a large coverage of semipolar and polar compounds, combining a minimally invasive sample treatment with different instrumental techniques. However, to obtain a comprehensive picture including highly lipophilic compounds, additional analysis using gas chromatography (GC) coupled to HRMS would be necessary. However, the observed co-occurrence of chemicals in sludge and humans in our experiments, as well as because of data crossing from different national monitoring studies, strongly suggests that wide-scope analysis of sludge can be used to obtain information on chemical exposure in human populations.

## Supporting Information Available

Summary of study population and sample collection (Section SI-1), summary of chemicals and materials (Section SI-2), detailed information regarding the sample treatment (Section SI-3), full details on instrumental analysis (Section SI-4), table containing chromatographic gradients (Table S1), figure with instrumental and data treatment workflow (Figure S1), detailed information regarding suspect screening data processing (Section SI-5), nontarget screening (Section SI-6), semiquantification strategy (Section SI-7), van Krevelen diagrams creation (Section SI-8), and table with analytical evidence leading to chemical identifications (Table S2) (PDF)
Complete list of chemicals evaluated in the suspect analysis (XLSX)
