# Supplementary material for: The Potential of Sewage Sludge to Predict and Evaluate the Human Chemical Exposome
Source: Environ Sci Technol Lett. 2021 Nov 12;8(12):1077–84. doi: 10.1021/acs.estlett.1c00848 (PMC9132361; doi:10.1021/acs.estlett.1c00848)
Supplement: Supplementary file 1 — ez1c00848_si_001.pdf [file ez1c00848_si_001.pdf]

SUPPORTING INFORMATION TO:

The potential of sewage sludge to predict and evaluate the human's chemical exposome

Gil-Solsona, Ruben[1]; Nika, Maria-Christina[2]; Bustamante, Mariona[3,4,5]; Villanueva, Cristina M.[3,4,5,6]; Foraster, Maria[3,4,5,7]; Cosin-Tomás, Marta[8]; Alygizakis, Nikiforos[2]; Gómez-Roig, Maria Dolores[9]; Llurba-Olive, Elisa[10,11]; Sunyer, Jordi[3,4,5]; Thomaidis, Nikolaos S.[2]; Dadvand, Payam[3,4,5]; Gago-Ferrero, Pablo[1,*]

[1]Department of Environmental Chemistry, Institute of Environmental Assessment and Water Research – Severo Ochoa Excellence Center (IDAEA), Spanish Council of Scientific Research (CSIC), Barcelona, Jordi Girona 18-26, 08034, Spain

[2]Laboratory of Analytical Chemistry, Department of Chemistry, National and Kapodistrian University of Athens, Panepistimiopolis Zografou, Athens, 15771, Greece

[3]ISGlobal, Barcelona, 08003, Spain

[4]Universitat Pompeu Fabra (UPF), Barcelona, 08003, Spain

[5]CIBER Epidemiología y Salud Pública (CIBERESP), Madrid, 28029, Spain

[6] IMIM (Hospital del Mar Medical Research Institute), Barcelona, Doctor Aiguader 88, 08003, Spain

[7] PHAGEX Research Group, Blanquerna School of Health Science, Universitat Ramon Llull (URL), Barcelona, Carrer de Padilla, 326, 08025, Spain

[8]Department of Human Genetics, McGill University, Research Institute of the McGill University Health Center, Montreal, 845 Sherbrooke St W, Quebec H3A 0G4, Canada

[9]BCNatal – Barcelona Center for Maternal Fetal and Neonatal Medicine (Hospital Sant Joan de Déu and Hospital Clínic), University of Barcelona, Esplugues de Llobregat, Passeig de Sant Joan de Déu, 2, 08950, Barcelona, Spain

[10]Maternal and Fetal Medicine Unit, Obstetrics and Gynecology Department, Sant Pau University Hospital, Barcelona , C. de Villarroel, 170, 08036, Spain.

[11]Development Network (SAMID), RD16/0022/0015, Instituto de Salud Carlos III, Madrid , Av. de Monforte de Lemos, 5, 28029, Spain

[*]Corresponding author: Pablo Gago-Ferrero
Tel: +34 934 00 61 00 E-mail: pablo.gago@idaea.csic.es

CONTENT:

SI-1 Study population and sample collection

SI-2: Chemicals and materials

SI-3: Sample treatment

SI-4: Instrumental analysis

Table S1: Chromatographic gradients in positive (left) and negative (right) ionization modes

Figure S1: Instrumental and data treatment workflow.

SI-5: Suspect screening data processing

SI-6: Non-target screening data processing

SI-7 Semi-quantification of the chemicals for which no reference standards were available

SI-8: van Krevelen diagrams

Table S2: Analytical evidences leading to chemical identifications

SI-1 Study population and sample collection

BiSC is a prospective population-based cohort study of pregnant women, their offspring and partners, that aims to identify early environmental and genetic causes of development and health from foetal life until young adulthood. Inclusion criteria are: singleton pregnancies, being 18 to 45 years old, residing in the Barcelona area, and being able to communicate in Spanish/Catalan. Placenta donors were European (78%) and Latinamerican (22%), with an age range from 29 to 36 years old. Serum donors were European (70%), Latinamerican (20%) and Arab (10%), with an age range from 33 to 42 years old. The study and all the consent forms were approved by the ethical committee of the Parc de Salut Mar (number:2018/8050/I).

SI-2: Chemicals and materials

Acetonitrile (ACN) (HPLC-grade), Methanol (HPLC-grade), Water (HPLC-grade), Ethyl acetate (HPLC-grade) and Formic acid (>99% purity) were purchased from Merck (Darmstadt, Germany). Distilled water was provided by a Milli-Q purification apparatus (Millipore, Bedford, MA, USA). Citric acid and Tri-sodium citrate were purchased from Scharlab (Barcelona, Spain). Citric acid solution 0.1M and Tri-sodium citrate-2-hydrate solution 0.1M were prepared in Milli-Q water. A buffer (pH:4) was prepared with 59% of citric acid solution and 41% citrate solution and mixed with ACN (1:1, v/v) for solid-liquid extractions. Analytical standards for target, suspect and non-target analysis were purchased from Sigma-Aldrich (Steinheim, Germany).

The digital sonifier, used for the extraction of sludge samples, was purchased from Fischer Scientific (Madrid, Spain). A Tissuelyzer LT from Qiagen (Barcelona, Spain) was used for the placenta extraction. The empty solid phase extraction (SPE) polypropylene tubes (6 mL), as well as the cartridge sorbent materials Sepra ZT (Strata-X), Sepra ZT- WCX (Strata-X-CW), ZT-WAX (Strata-X-AW) and the frits (20 µm, 6 mL) were obtained from Phenomenex (Torrance, USA). The Isolute ENV+ sorbent material was obtained from Biotage (Ystrad Mynach, UK). Tissuelyzer tubes

were purchased from Deltalab (Barcelona, Spain) and zirconium beads from VWR International Eurolabs (Barcelona, Spain).

SI-3: Sample treatment

Blood: Serum and plasma samples, stored at -80°C, were thawed at room temperature. Then, an aliquot of 200 µL was transferred to an Eppendorf vial (2 mL) and 600 µL of ACN were added. Sample was vigorously shaken by hand in order to avoid clotting and further vortexed during 15 seconds. Then, samples were centrifuged (10 min, 10000 rpm) and the supernatant was transferred to a chromatographic glass vial for instrumental analysis.

Placenta: Placenta samples, stored at -80°C, were thawed at room temperature. The sample (200 mg) was transferred into a tissuelyzer tube containing 1g of zirconium beads. Then, 1 mL of a mixture of citrate buffer: ACN (1:1, v/v) was added, and the sample was extracted with the Tissuelyzer (2 min, 50 Hz). Then, the extract was centrifuged (10 min, 10000 rpm) and the supernatant was transferred into a glass tube. This process was repeated by triplicate, to ensure a complete extraction. Sample extracts were led to approximately half of the initial volume (under a gentle N_2 current) to evaporate the ACN and then added to 100mL of Milli-Q water for further SPE clean-up. Home-made SPE cartridges (described elsewhere[1]) were previously conditioned with 6 mL MeOH and 6 mL Milli-Q water. The solutions were loaded in the cartridge at a constant flow (2 drops per second). Finally, the elution was carried out with 4 mL of Ethyl acetate:Methanol (1:1) 2% NH_3 and 2 mL of Ethyl acetate: Methanol (1:1) 1.8% Formic acid. Then, the extracts were led to dryness and reconstituted with 0.2 mL of MeOH:H_2O (1:1, v/v) for instrumental analysis.

Sludge: Sludge was freeze-dried after sampling. Then, 1g was weighed in a 15mL falcon tube. After that, 6.7 mL ACN were added and sonicated with a Digital Sonifier (Amplitude 40%, 60s).

The solution was then centrifuged (5000 rpm, 10 min) and the supernatant was stored in a 50mL falcon tube. This process was repeated with (a) 6.7 mL of ACN:H_2O (1:1) and (b) 6.7 mL of H_2O for a complete extraction. The supernatants were led to half of the initial volume with a gentle current of N_2 and added to 90mL of Milli-Q water for SPE extraction. SPE was conducted in the same manner than the previously described for placenta samples.

Blank samples: Blank samples were prepared following the same protocol for each matrix (specific blanks for serum, placenta and sludge) without the addition of sample (procedural blanks). The chemicals detected in blank samples were carefully quantitated with a calibration curve in HPLC-grade solvents. Only when concentration found in the sample was at least 10-times higher than those found in the blanks, the chemical was considered detected.

Standard addition quantification method: In order to counteract potential matrix effects, matrix-matched calibration curves were prepared at different concentration levels (ranging from 0.01 to 500 ng/mL in the final extracts). They were used in the (semi)quantification of the chemicals identified in serum, placenta and sludge samples.

SI-4: Instrumental analysis

Target analysis was carried out using an UHPLC/QTOF-MS system, equipped with a UHPLC apparatus (Dionex UltiMate 3000 RSLC, Thermo Fisher Scientific, Dreieich, Germany), consisting of a solvent rack degasser, auto-sampler, a binary pump with solvent selection valve and a column oven coupled to the QTOF-MS mass analyzer (Maxis Impact, Bruker Daltonics, Bremen, Germany). Instrumental parameters are described elsewhere[2].

For suspect and non-target screening, instrumental analysis was performed using an UHPLC system coupled to a Q-Exactive Orbitrap mass analyser (Thermo Fisher Scientific, Dreieich,

Germany). The chromatographic column was a Cortecs C18 (2.1x100 mm, 2.7µm) from Waters, preceded by a guard column of the same packaging material, kept at 40°C. In positive ionization mode, the aqueous phase consisted of H_2O with 0.1% of formic acid, and the organic phase was MeOH with 0.1% of formic acid. In negative ionization mode, the aqueous phase consisted of H_2O 5mM ammonium acetate, and the organic phase was MeOH 5mM ammonium acetate. The elution gradients in both ionization modes are shown in **Table S1**. The flow rate was constant (0.3 mL/min) and the injection volume was set to 5 µL of sample. The Orbitrap system was equipped with an electrospray ionization interphase (ESI), operating at 3000 V in positive and 2800V in negative ionization modes, 350°C capillary temperature, 40 sheath gas flow, 10 auxiliary gas flow, 100 of maximum spray current, 350°C probe heater temperature and 60 S-Lens RF level.

Two different acquisitions were carried out for each sample, one in data dependent (DDA) and other in data independent (DIA) acquisition. In both cases, full scan mass spectra were recorded over the range of 67-1000 *m/z* with a resolving power of 60000. MS/MS experiments for DDA were conducted for the 5 most intense ions in each scan at resolving power of 30000 at 35eV collision energy, excluding the previously selected ions for MS/MS acquisitions for the next 30 seconds. For DIA, 25eV collision energy was applied.

Table S1: Chromatographic gradients in positive (left) and negative (right) ionization modes.

Time (min)	% A	%B
0	95	5
7	25	75
10	0	100
17	0	100
18	95	5

Time (min)	% A	%B
0	95	5
3	50	50
6	10	90
13	0	100
17	0	100
18	95	5

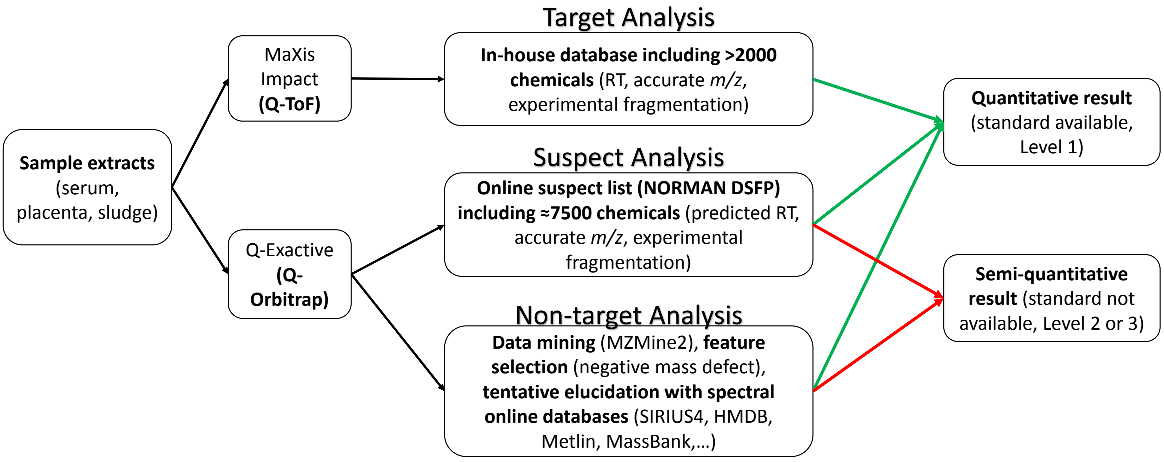

Figure S1: Instrumental and data treatment workflow.

SI-5: Suspect screening data processing

For suspect screening, data obtained from the injections in the Q-Exactive instrument (in DIA) was transformed from proprietary (*.raw) to generic (*.mzML) format using ProteWizard software[3]. DIA data was split in two files (low energy and high energy functions) using the Digital Sample Freezing Platform (DSFP) Split tool[4]. Then, data was uploaded in the main DSFP platform and suspect screening was conducted for chemicals with available experimental MS/MS data (suspect *lists EXPHRMSMSAVALPOS* and *EXPHRMSMSAVALNEG*) as described elsewhere[4].

All the positive findings obtained in the aforementioned manner were manually checked for MS/MS spectra in the DDA data. When MS/MS was available, the experimental spectra were compared with those present in different databases (MassBank[5], MassBank of North America[6], Metlin[7], HMDB[8] and mzCloud) in order to keep (or discard) the identification via manual comparison. When spectra similarity was high, the compound was kept as tentatively identified. This strategy is highly restrictive, so the possibility of false negative assignations is high, as chemicals without MS/MS acquisition are discarded. However, we put the focus on maximizing the avoidance of false positive results (comparing MS/MS data with spectral libraries, as well as their experimental retention time with the one predicted[9]).

For 15 out of the 28 tentatively identified chemicals, commercial analytical standards were available and therefore purchased for confirmation. Their identity was fully confirmed in all cases, showing the good performance of the approach. The other 13 chemicals remained as tentatively identified with a confidence level of 2A according to the hierarchical levels described by Schymanski et al[10], since the similarity of the MS/MS spectra with those corresponding to the aforementioned databases was confirmed and the experimental RT fitted well with the predicted one but reference standards were not available.

SI-6: Non-target screening data processing

For non-target screening, data obtained from the injections in the Q-Exactive instrument (in DDA) was transformed from proprietary (*.raw) to generic (*.mzML) following the aforementioned process. Data was imported into MZMine2[10] and data mining was performed with the following parameters:

- Mass Detection:
    - MS Level: 1 and 2
    - Noise level: $1.0E^3$ (in both POS and NEG ionization modes).
- ADAP chromatogram builder:
    - MS level: 1
    - Min group size in # of scans: 5
    - Group intensity threshold: $1.0E^5$
    - Min highest intensity: $1.0E^5$
    - *m/z* tolerance: 0.001 m/z or 3 ppm
- Chromatogram deconvolution
    - Algorithm: Wavelets (ADAP)
        - S/N threshold: 10

        - S/N estimator: Intensity window SN
        - Min feature height: 1.0E[5]
        - Coefficient/area threshold: 55
        - Peak duration range: 0.00-1.00
        - RT wavelength range 0.00-1.00
    - *m/z* center calculation: MEDIAN
    - *m/z* range for MS2 scan pairing (Da): Checked, 0.001
    - RT range for MS2 scan pairing (min): Checked, 0.15
- Isotope peak grouper:
    - *m/z* tolerance: 0.001 *m/z* or 3 ppm
    - Retention time tolerance: 0.08 Absolute (min)
    - Maximum charge: 2
    - Representative isotope: Lowest *m/z*
- Peak filter:
    - Keep only features with MS/MS scan: Checked
- Join Aligner:
    - *m/z* tolerance: 0.001 *m/z* or 3 ppm
    - Weight for *m/z*: 60
    - Retention time tolerance: 0.15 absolute (min)
    - Weight for RT: 40
- Peak finder:
    - Intensity tolerance: 10%
    - *m/z* tolerance: 0.001 *m/z* or 3 ppm
    - Retention time tolerance: 0.15 absolute (min)
- Export for SIRIUS
- Export to CSV file

For molecular formula assignation, SIRUS 4.7.0[11] was used with the following parameters:

- SIRIUS – Molecular Formula Assignation:
    - Instrument: Orbitrap
    - MS/MS isotope scorer: SCORE
    - MS2 MassDev (ppm): 3
    - Candidates: 10
    - Candidates per ion: 1
    - Consider only formulas in DBs: All
    - Possible Ionizations:
        - POS: M+H, M+Na, M+K
        - NEG: M-H, M+Cl
- ZODIAC: Network-based improvement of SIRIUS molecular formula ranking:
        - Parameters by default
- CSI:FingerID – Structure Elucidation
        - Search in DBs: All
        - Fallback adducts:
            - POS: M+H
            - NEG: M-H
- CANOPUS – Compound Class Prediction

SIRIUS4 software generates reliable molecular formulas and tentative structural identifications of unknown compounds by using the available information on isotope pattern, accurate mass and MS/MS information. For features with an observed mass defect (manually filtered from the excel file created with all the features obtained with MZmine2), the tentative identifications were manually checked and, when available, compared with the spectra observed in the

previously mentioned databases: MassBank[5], MassBank of North America[6], Metlin[7], HMDB[8] and mzCloud. These chemicals whose standards were available to purchase were confirmed at level 1, according to Schymanski[10]. The rest were identified at Level 2 or 3[10], depending on the availability of their MS/MS spectra in the databases.

SI-7 Semi-quantification of the chemicals for which no reference standards were available

For the identified chemicals for which no reference standards were available (Level 2 or Level 3[10]), semi-quantification was conducted by using the most similar chemical included in the calibration curves prepared for the rest of the chemicals investigated in the study. For both the newly identified chemicals as well as the xenobiotics included in the calibration curves the ionization efficiency was estimated[12,13]. In this manner the compound used to semi-quantify was selected and semi-quantification was performed based on peak area.

SI-8: van Krevelen diagrams

The van Krevelen diagrams were built based on the molecular formulae assigned by SIRIUS4 to all the features for which MS/MS data was available. Data was imported to R and treated with the ftmsRanalysis package using the default script provided by the authors[14]. Features were represented according to their H/C and O/C ratios. Then, they were divided in regions defined as compositional spaces[15] (e.g. lipid-like, protein-like). In the present study we use these diagrams to obtain an overall picture of which types of chemicals are common (or not common) among the matrices of interest. However, it should be emphasized that the labels of these van Krevelen regions do not reflect the complex nature of the whole set of features contained therein[16].

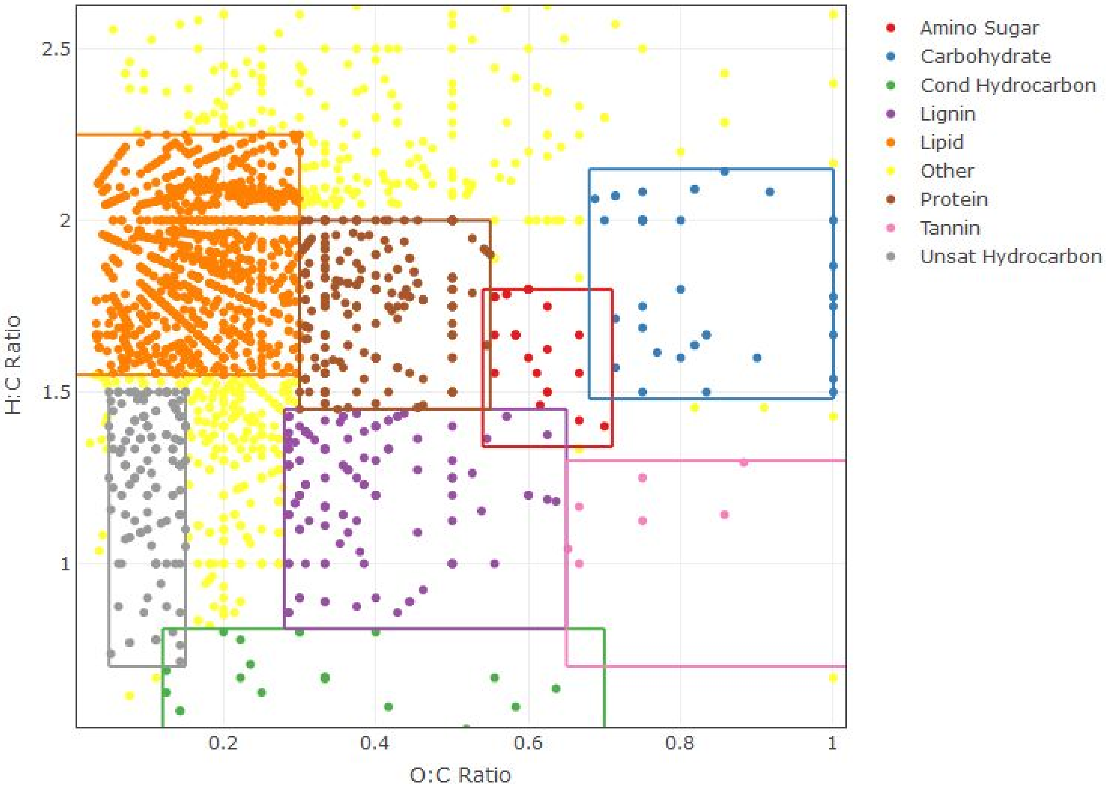

Figure S2: van Krevelen diagram representing the common features (for which a molecular formula could be assigned) among sludge and human tissue/biofluid

Table S2: Analytical evidences leading to chemical identifications

Identity	Confidence level	LoD* serum (ng/mL)	LoD* placenta (ng/g)	LogP **	Accurate mass $[M+H]^+/[M-H]^-$	Identification proofs
**Tris(chloropropyl)phosphate**	Level 1	0.05	0.10	2.0	$[M+H]^+$ 327.0081	Accurate mass, RT confirmed with standard, MS/MS fragment ions (*m/z:* 174.9924, 98.9842), distinctive isotopic pattern
**1,7-Dimethylxanthine**	Level 2	-	-	-0.2	$[M+H]^+$ 181.0717	Accurate mass, agreement with predicted RT, MS/MS fragment ions (*m/z:* 124.0507, 142.0614)
**Theobromine**	Level 1	0.01	0.01	-0.8	$[M+H]^+$ 181.0717	Accurate mass, RT confirmed with standard, MS/MS fragment ions (*m/z*: 138.0664, 110.0714)
**Theophylline**	Level 1	0.01	0.01	0	$[M+H]^+$ 181.0717	Accurate mass, RT confirmed with standard, MS/MS fragment ions (*m/z*: 124.0508, 142.0614)
**Lenticin**	Level 2	-	-	2.9	$[M+H]^+$ 247.1436	Accurate mass, agreement with predicted RT, MS/MS fragment ions (*m/z:* 188.0708, 146.0601, 60.0811)
**Caffeine**	Level 1	0.01	0.01	-0.1	$[M+H]^+$ 195.0873	Accurate mass, RT confirmed with standard, MS/MS fragment ions (*m/z*: 138.0663, 110.0713)
**Endothall**	Level 1	0.01	0.05	3.0	$[M-H]^-$ 185.0455	Accurate mass, RT confirmed with standard, MS/MS fragment ion (*m/z*: 141.0557)
**Denatonium**	Level 1	1.5	4.9	4.1	$[M+H]^+$ 325.2273	Accurate mass, RT confirmed with standard, MS/MS fragment ions (*m/z*: 233.1646, 86.0964)
**Benzotriazole**	Level 1	0.2	0.2	0.4	$[M+H]^+$ 120.0557	Accurate mass, RT confirmed with standard, MS/MS fragment ions (*m/z*: 92.0496, 65.0388)
**Dibenzylamine**	Level 2	-	-	2.7	$[M+H]^+$ 198.1279	Accurate mass, agreement with predicted RT, MS/MS fragment ions (*m/z*: 106.0653, 91.0544)
**Tributyl phosphate**	Level 2	-	-	2.9	$[M+H]^+$ 267.1716	Agreement with Accurate mass, agreement with predicted RT, MS/MS fragment ions (*m/z*: 155.0468, 98.9842)

**Triphenylphosphine oxide**	Level 1	0.01	0.02	2.8	$[M+H]^+$ 279.0934	Accurate mass, agreement with predicted RT, MS/MS fragment ions (*m/z*: 201.0465, 173.0521)
**Triphenyl phosphate**	Level 1	0.01	0.02	2.0	$[M+H]^+$ 327.0777	Accurate mass, RT confirmed with standard, MS/MS fragment ions (*m/z*: 233.0361, 153.0699)
**2-hydroxybenzothiazole**	Level 1	0.1	0.2	1.8	$[M-H]^-$ 152.0153	Accurate mass, RT confirmed with standard, MS/MS fragment ion (*m/z*: 124.0206)
**2-benzothiazolesulfonic acid**	Level 1	0.1	0.1	1.5	$[M-H]^-$ 213.9636	Accurate mass, agreement with predicted RT, MS/MS fragment ions (*m/z*: 150.0019, 134.0071), distinctive isotopic pattern
**DEET**	Level 1	0.01	0.02	2.0	$[M+H]^+$ 192.1381	Accurate mass, RT confirmed with standard, MS/MS fragment ions (*m/z*: 119.0492, 100.0757, 72.0445)
**N,N-Dimethyldodecylamine-N-oxide**	Level 2	-	-	5.3	$[M+H]^+$ 230.2473	Accurate mass, RT confirmed with standard, MS/MS fragment ion (*m/z*: 58.0655)
**Panthenol**	Level 1	1.7	1.7	-0.9	$[M+H]^+$ 206.1389	Accurate mass, RT confirmed with standard, MS/MS fragment ions (*m/z*: 105.0698)
**Bis(2-ethylhexyl)amine**	Level 2	-	-	6.1	$[M+H]^+$ 242.2839	Accurate mass, agreement with predicted RT, MS/MS fragment ions (*m/z*: 85.1013, 71.0857, 57.0703)
**N-(2-hydroxyethyl)-Tetradecanamide**	Level 2	-	-	5.1	$[M+H]^+$ 272.2582	Accurate mass, RT confirmed with standard, MS/MS fragment ions (*m/z*: 255.2313, 71.0846)
**Lauryl diethanolamine**	Level 1	0.01	0.01	4.5	$[M+H]^+$ 274.2734	Accurate mass, RT confirmed with standard, MS/MS fragment ions (*m/z*: 256.2637, 106.0863, 88.0758)
**Benzododecinium**	Level 1	1.0	1.0	7.4	$[M+H]^+$ 304.2996	Accurate mass, RT confirmed with standard, MS/MS fragment ions (*m/z*: 212.2374, 91.0543, 58.0655)
**PFOS**	Level 1	0.01	0.01	5.0	$[M-H]^-$ 498.9297	Accurate mass, RT confirmed with standard, MS/MS fragment ions (*m/z*: 168.9896, 98.9561, 79.9575)

**PFHpS**	Level 1	0.01	0.01	4.3	$[M-H]^-$ 448.9323	Accurate mass, RT confirmed with standard, MS/MS fragment ions (*m/z*: 168.9896, 98.9561, 79.9575)
**PFBS**	Level 1	0.01	0.01	2.3	$[M-H]^-$ 298.9420	Accurate mass, RT confirmed with standard, MS/MS fragment ions (*m/z*: 168.9896, 98.9561, 79.9575)
**6:2 FTS**	Level 1	0.01	0.01	3.9	$[M-H]^-$ 426.9667	Accurate mass, RT confirmed with standard, MS//MS fragment ions (*m/z*: 406.9615, 386.9556, 80.9653)
**PFOA**	Level 1	0.01	0.01	4.9	$[M-H]^-$ 412.9655	Accurate mass, RT confirmed with standard, MS/MS fragment ions (*m/z*: 168.9897, 118.9929, 78.9592)
**PFHxS**	Level 1	0.01	0.01	3.6	$[M-H]^-$ 398.9358	Accurate mass, RT confirmed with standard, MS/MS fragment ions (*m/z*: 168.9896, 98.9561, 79.9575)
**Bupivacaine**	Level 2	-	-	3.4	$[M+H]^+$ 298.2276	Accurate mass, agreement with predicted RT, MS/MS fragment ions (*m/z*: 140.1434, 98.0965)
**Ciprofloxacin**	Level 1	0.2	0.2	-1.1	$[M+H]^+$ 332.1408	Accurate mass, RT confirmed with standard, MS/MS fragment ions (*m/z*: 314.1299, 288.1503, 245.1081)
**Amoxicillin**	Level 1	0.5	0.5	-2.0	$[M+H]^+$ 366.1121	Accurate mass, RT confirmed with standard, MS/MS fragment ions (*m/z*: 208.0421, 114.0012)
**Indole-3-acetic acid**	Level 1	0.1	0.2	1.4	$[M+H]^+$ 176.0706	Accurate mass, RT confirmed with standard, MS/MS fragment ions (*m/z*: 130.0652, 103.0541)
**Dibutyl phthalate**	Level 2	-	-	4.7	$[M+H]^+$ 279.1589	Accurate mass, agreement with predicted RT, MS/MS fragment ions (*m/z*: 149.0234, 57.0702)
**Monobuthyl phthalate**	Level 2	-	-	3.1	$[M+H]^+$ 223.0965	Accurate mass, agreement with predicted RT, MS/MS fragment ions (*m/z*: 149.0234, 57.0702)
**4-Ethoxyethylbenzoate**	Level 2	-	-	3.2	$[M+H]^+$ 195.1014	Agreement with Accurate mass, agreement with predicted RT, MS/MS fragment ions (*m/z*: 149.0598, 121.0285, 95.0492)

**1,3-Diphenylguanidine**	Level 2	-	-	2.4	$[M+H]^+$ 212.1180	Agreement with Accurate mass, agreement with predicted RT, MS/MS fragment ions (*m/z*: 195.0918, 119.0605, 94.0652)
**Nicotine**	Level 1	0.1	0.1	1.2	$[M+H]^+$ 163.1231	Accurate mass, RT confirmed with standard, MS/MS fragment ions (*m/z*: 132.0807, 106.0651, 84.0808)
**Cotinine**	Level 1	0.1	0.1	-0.3	$[M+H]^+$ 177.1021	Accurate mass, RT confirmed with standard, MS/MS fragment ions (*m/z*: 98.0599, 80.0495)
**Acesulfame**	Level 1	0.1	0.1	-0.3	$[M-H]^-$ 161.9862	Accurate mass, RT confirmed with standard, MS/MS fragment ions (*m/z*: 82.0300), distinctive isotopic pattern
**Methylsulfonyl-1,3-thiazol-2-ylamine**	Level 3	-	-	-0.8	$[M+H]^+$ 178.9946	Accurate mass, fragment ions in MS/MS (*m/z*: 164.9788, 105.9416, 78.9849)
**1-Benzothiophene-2-Sulfonamide**	Level 3	-	-	1.8	$[M+H]^+$ 213.9995	Accurate mass, fragment ions in MS/MS (*m/z*: 181.9729, 141.0005, 95.0492)
**Benzophenone**	Level 1	0.05	0.05	3.4	$[M+H]^+$ 183.0803	Accurate mass, RT confirmed with standard, MS/MS fragment ions (*m/z*: 105.0336, 95.0492)

*LoD: limit of detection (LODs were estimated as the lower concentration that could be observed in the matrix-matched calibration curves), **LogP values have been retrieved from Pubchem database (https://pubchem.ncbi.nlm.nih.gov/)

References:

1. Gago-Ferrero P, Schymanski EL, Bletsou AA, Aalizadeh R, Hollender J, Thomaidis NS. Extended Suspect and Non-Target Strategies to Characterize Emerging Polar Organic Contaminants in Raw Wastewater with LC-HRMS/MS. *Environ Sci Technol*. 2015;49(20):12333-12341. doi:10.1021/acs.est.5b03454
2. Gago-Ferrero P, Bletsou AA, Damalas DE, Aalizadeh R, Alygizakis NA, Singer HP, Hollender J, Thomaidis NS. Wide-scope target screening of >2000 emerging contaminants in wastewater samples with UPLC-Q-ToF-HRMS/MS and smart evaluation of its performance through the validation of 195 selected representative analytes. *J Hazard Mater*. 2020;387:121712. doi:10.1016/j.jhazmat.2019.121712
3. Chambers MC, MacLean B, Burke R, Amodei D, Ruderman DL, Neumann S, Gatto L, Fischer B, Pratt B, Egertson J, Hoff K, Kessner D, Tasman N, Shulman N, Frewen B, Baker TA, Brusniak MY, Paulse C, Creasy D, FlashnerL, et al. A cross-platform toolkit for mass spectrometry and proteomics. *Nat Biotechnol*. 2012;30(10):918-920. doi:10.1038/nbt.2377
4. Alygizakis NA, Oswald P, Thomaidis NS, Schymanski EL, Aalizadeh R, Schulze T, Oswaldova M, Slobodnik J. NORMAN digital sample freezing platform: A European virtual platform to exchange liquid chromatography high resolution-mass spectrometry data and screen suspects in “digitally frozen” environmental samples. *TrAC - Trends Anal Chem*. 2019;115:129-137. doi:10.1016/j.trac.2019.04.008
5. MassBank | European MassBank (NORMAN MassBank) Mass Spectral DataBase.. http://massbank.eu/MassBank/Index, last accessed November 2021
6. MassBank of North America. https://mona.fiehnlab.ucdavis.edu/, last accessed November 2021
7. Guijas C, Montenegro-Burke JR, Domingo-Almenara X, Palermo A, Warth B, Hermann G, Koellensperger G, Huan T, Uritboonthai W, Aisporna AE, Wolan DW, Spilker ME, Benton

HP, Siuzdak G. METLIN: A Technology Platform for Identifying Knowns and Unknowns. *Anal Chem*. 2018;90(5):3156-3164. doi:10.1021/acs.analchem.7b04424

8. Wishart DS, Tzur D, Knox C, Eisner R, Guo AC, Young N, Cheng D, Jewell K, Arndt D, Sawhney S, Fung C, Nikolai L, Lewis M, Coutouly MA, Forsythe I, Tang P, Shrivastava S, Jeroncic K, Stothard P, Amegbey G, et al. HMDB: The human metabolome database. *Nucleic Acids Res*. 2007;35(SUPPL. 1). doi:10.1093/nar/gkl923

9. Aalizadeh, Reza; Alygizakis, Nikiforos; Schymanski, Emma; Krauss, Martin; Schulze, Tobias; Ibáñez, Maria; McEachran, Andrew D.; Chao, Alex; Williams, Antony John; Gago-Ferrero, Pablo; Covaci, Adrian; Moschet, Christoph; Young, Thomas; Hollender, Juliane; N. Development and Application of Liquid Chromatographic Retention Time Indices in HRMS-based Suspect and Non-target Screening. *Anal Chem*. 2021;93(33):11601-11611.

10. Pluskal T, Castillo S, Villar-Briones A, Orešič M. MZmine 2: Modular framework for processing, visualizing, and analyzing mass spectrometry-based molecular profile data. *BMC Bioinformatics*. 2010;11(1):395. doi:10.1186/1471-2105-11-395

11. Dührkop K, Fleischauer M, Ludwig M, Aksenov AA, Melnik A V., Meusel M, Dorrestein PC, Rousu J, Böcker S. SIRIUS 4: a rapid tool for turning tandem mass spectra into metabolite structure information. *Nat Methods*. 2019;16(4):299-302. doi:10.1038/s41592-019-0344-8

12. Aalizadeh R, Panara A, Thomaidis NS. Development and Application of a Novel Semi-quantification Approach in LC-QToF-MS Analysis of Natural Products. *J Am Soc Mass Spectrom*. 2021;32(6):1412-1423. doi:10.1021/jasms.1c00032

13. Alygizakis N, Galani A, Rousis NI, Aalizadeh R, Dimopoulos MA, Thomaidis NS. Change in the chemical content of untreated wastewater of Athens, Greece under COVID-19 pandemic. *Sci Total Environ*. 2021;799:149230. doi:10.1016/J.SCITOTENV.2021.149230

14. Bramer LM, White AM, Stratton KG, Thompson AM, Claborne D, Hofmockel K, McCue LA. ftmsRanalysis: An R package for exploratory data analysis and interactive

visualization of FT-MS data. Schneidman-Duhovny D, ed. *PLOS Comput Biol*. 2020;16(3):e1007654. doi:10.1371/journal.pcbi.1007654

15. Minor EC, Steinbring CJ, Longnecker K, Kujawinski EB. Characterization of dissolved organic matter in Lake Superior and its watershed using ultrahigh resolution mass spectrometry. *Org Geochem*. 2012;43:1-11. doi:10.1016/j.orggeochem.2011.11.007

16. Sanchís J, Jaén-Gil A, Gago-Ferrero P, Munthali E, Farré MJ. Characterization of organic matter by HRMS in surface waters: Effects of chlorination on molecular fingerprints and correlation with DBP formation potential. *Water Res*. 2020;176:115743. doi:10.1016/j.watres.2020.115743
